# Measles a Second Time in the Same Patients

**Published:** 1858-07

**Authors:** D. W. Young

**Affiliations:** Aurora, Ill.


ARTICLE IV.

### MEASLES A SECOND TIME IN THE SAME PATIENTS.

BY D. W. YOUNG, M. D., OF AURORA, ILL.

PROF. W. H. BYFORD, M. D.,

DEAR SIR,—We are having quite an extensive epidemic of measles in this city, at this time. They are mostly of a mild type, and quite amenable to treatment. In some of the cases, however, we have more or less pneumonic complications. I believe our cases, thus far, have all recovered, at least, I have heard of no deaths since the commencement of the epidemic.

The principal point of interest in these cases is the large number of persons that are having measles for the second time. I have often treated patients, sick with measles, who have declared that they had had measles before. Usually I have been unacquainted with their previous histories, and consequently paid but little attention to their statements. During the present epidemic I have had an opportunity of investigating this point more closely. I have myself met four cases during this epidemic, in persons that I know had had the measles before. Three of these patients I had attended when sick with measles, in the later part of December, 1853; the other one in February, 1854. Two of the patients had them severely the first time, and all of them very distinctly. The two patients that had them the worst the first time, are under treatment at the present time, and are having them comparatively light; while one of those who had them light before, has had them very severely this time. The eruption in all these cases, in both attacks, has been free, distinct and unmistakeable.

My friend and former partner, A. Hard, M. D., of this city, also informs me that he has met with quite a number of cases in persons who had had them before. One in his own family, a little daughter, who had them in 1854, and was then very sick, and came near dying with them, as I can testify, as I treated her in the earlier part of her attack myself, while her father was absent from the city. In her first attack, she had unusually severe pneumonic complications; so much so, that her father, who is one of *veratrum viride* eulogizers, represented her case in connection with several others, to the *Iowa Medical and Surgical Journal*, on the use of veratrum viride. She has had measles again severely and distinctly within a few days.

Another case that Dr. Hard reports is that of Rev. Mr. Bull, a very intelligent Congregational clergyman of this city, whom the Doctor treated a short time, while sick with measles. Mr. Bull declared that he had the measles while residing at the east, and after his recovery wrote to his mother, and among other things informed her that he was just recovering from a severe attack of measles. The old mother very affectionately informed him, by return of mail, that there must be some mistake; that he had the measles severely several years ago, and that she should doubt the qualifications of his western physician.

The Doctor, as well as several of our other physicians, report quite a number of other cases where the testimony is quite as clear as in the above cases. In conclusion, my opinion is that measles occur a second time in the same person, much oftener than our authorities and lecturers admit.

AURORA, JUNE 12, 1858.

